# Supplementary material for: Richness for Tumor-Infiltrating B-Cells in the Oral Cancer Tumor Microenvironment Is a Prognostic Factor in Early-Stage Disease and Improves Outcome in Advanced-Stage Disease
Source: Cancers (Basel). 2025 Jan 1;17(1):113. doi: 10.3390/cancers17010113 (PMC11719715; doi:10.3390/cancers17010113)
Supplement: Supplementary file 1 [file cancers-17-00113-s001.zip › Supplementary Figure S1.pdf]

Supplementary Figure S1

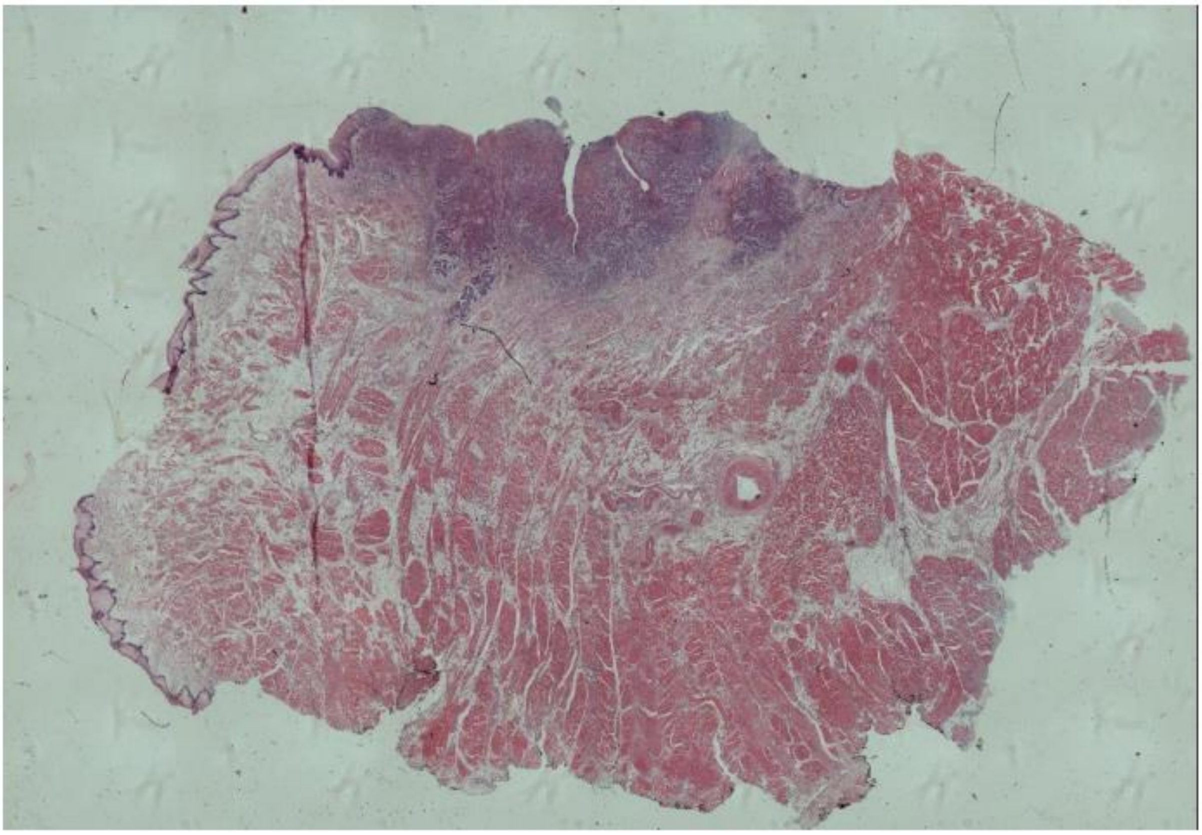

Supplementary Figure 1A. Example of an HE-stained slide with the tumor surrounded by a cuff of tumor-free tissue.

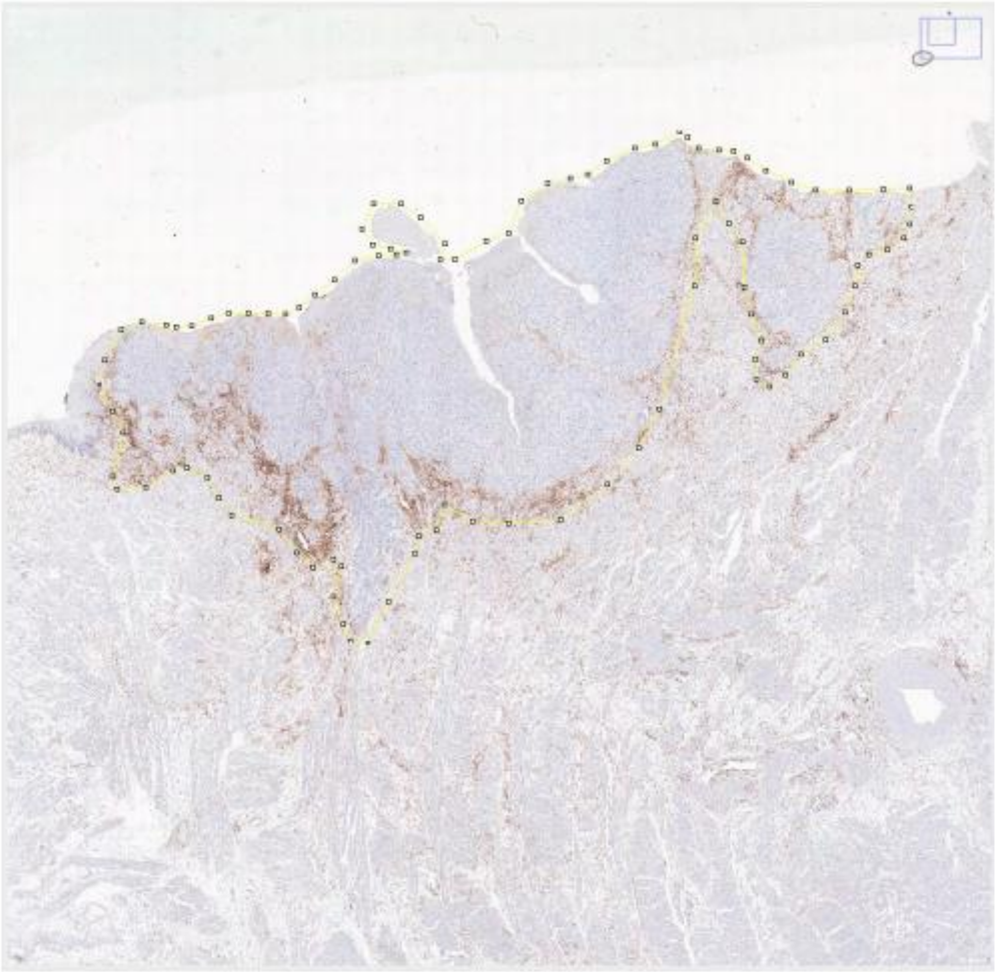

Supplementary Figure 1B. Example of the delineation (yellow line with dots) of the tumor area (consisting of the tumor with a cuff of tumor-free stroma).
