# Supplementary figures and images for: Richness for Tumor-Infiltrating B-Cells in the Oral Cancer Tumor Microenvironment Is a Prognostic Factor in Early-Stage Disease and Improves Outcome in Advanced-Stage Disease
Source: Cancers (Basel). 2025 Jan 1;17(1):113. doi: 10.3390/cancers17010113 (PMC11719715; doi:10.3390/cancers17010113)

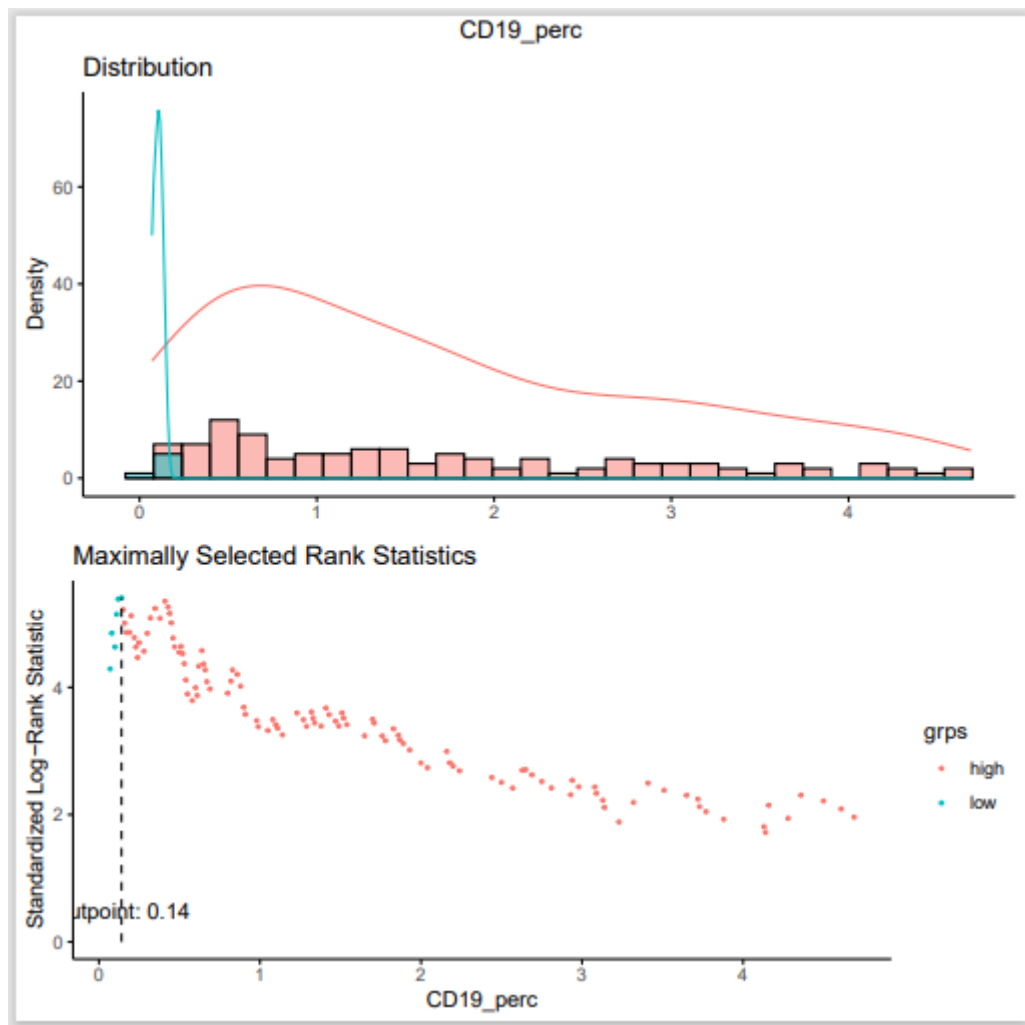

Supplement: Supplementary file 1 [file cancers-17-00113-s001.zip › Supplementary Figure S2.pdf]

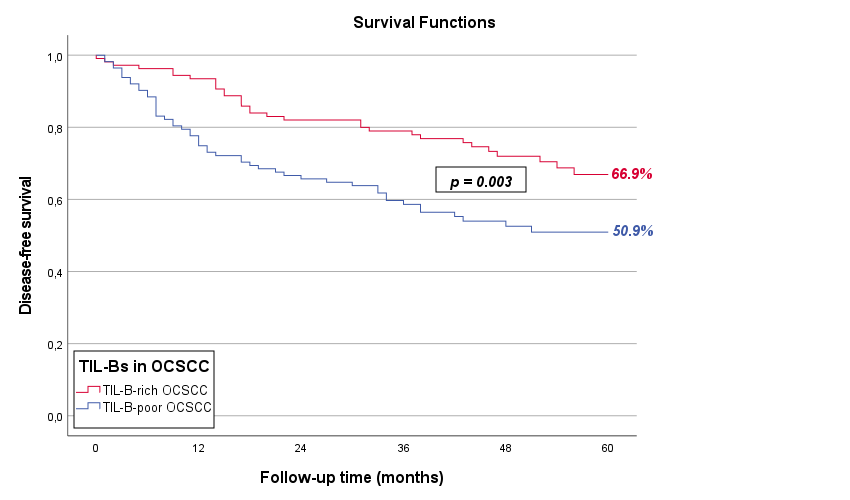

Supplement: Supplementary file 1 [file cancers-17-00113-s001.zip › Supplementary Figure S3A.tif]

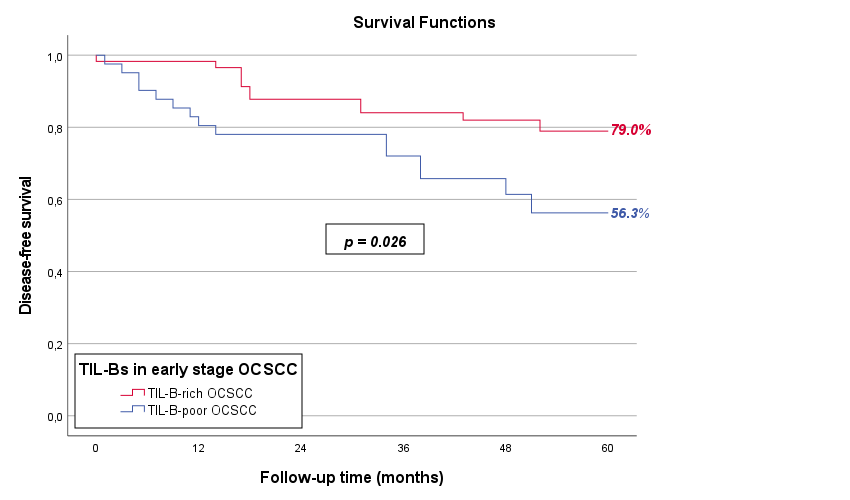

Supplement: Supplementary file 1 [file cancers-17-00113-s001.zip › Supplementary Figure S3B.tif]

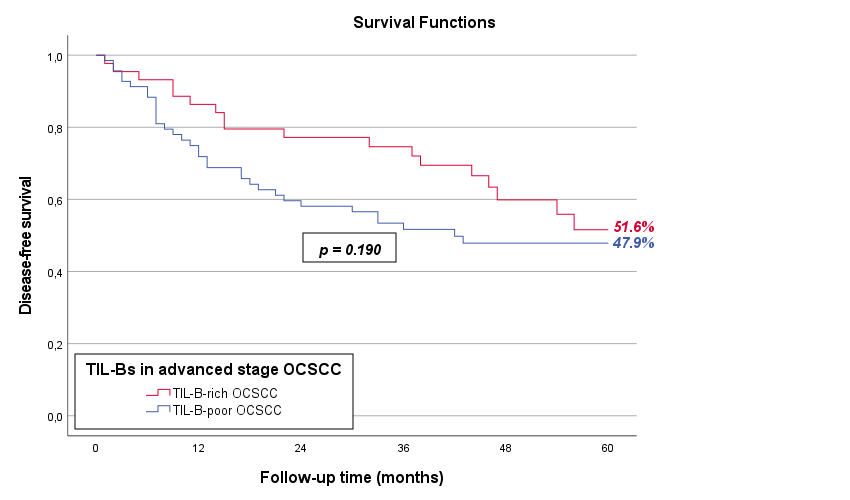

Supplement: Supplementary file 1 [file cancers-17-00113-s001.zip › Supplementary Figure S3C.tif]
